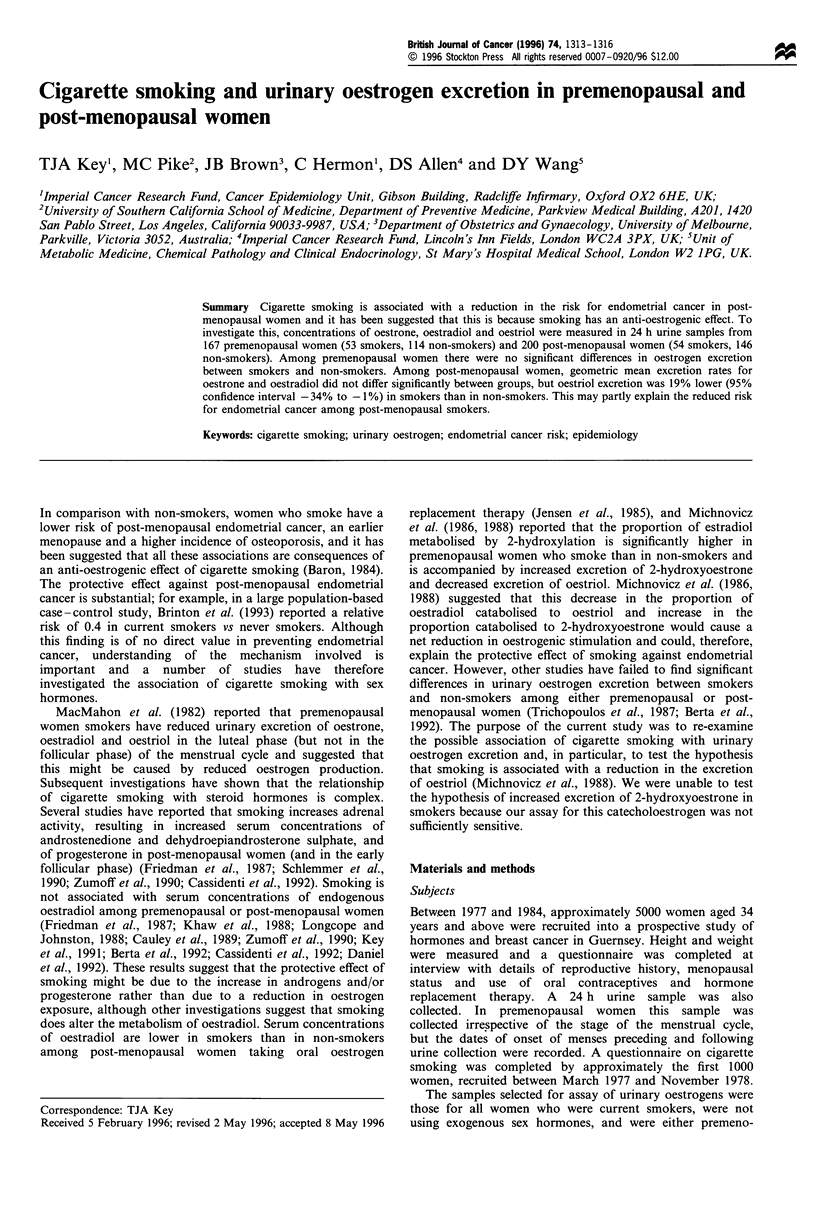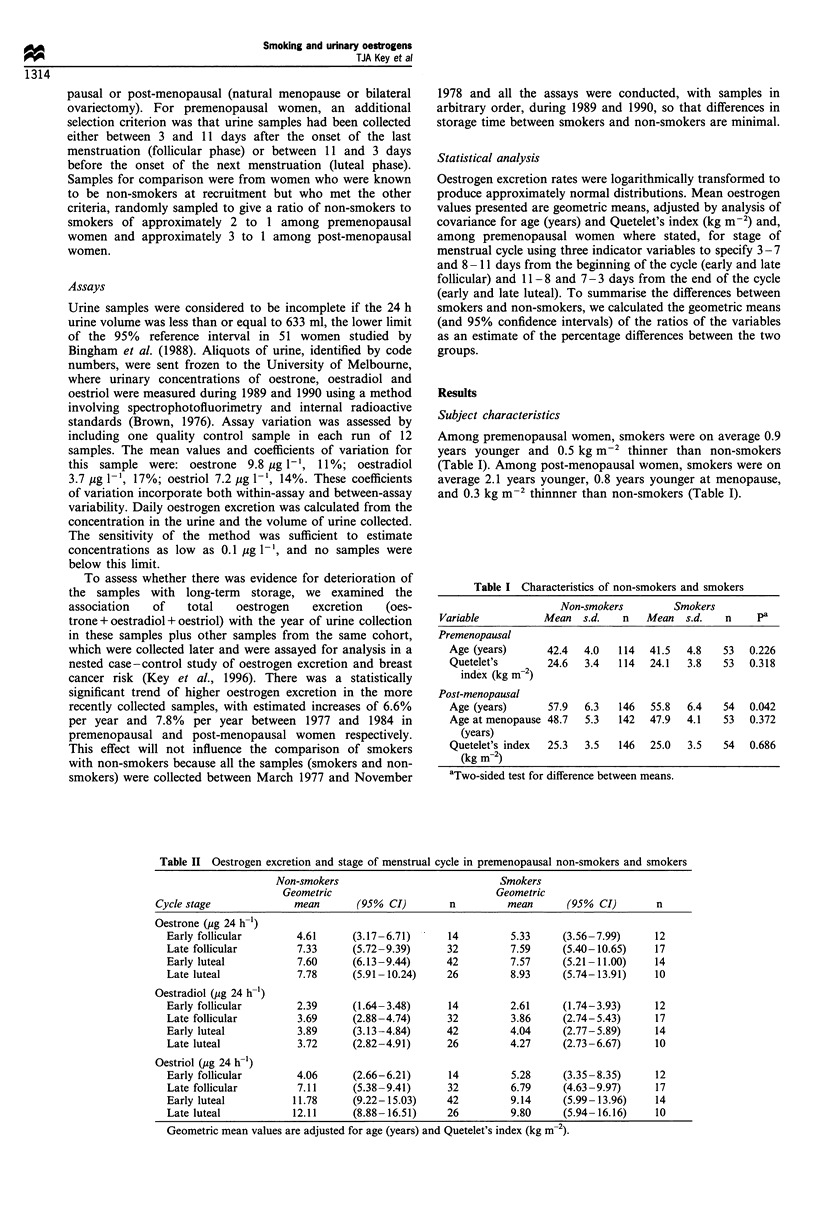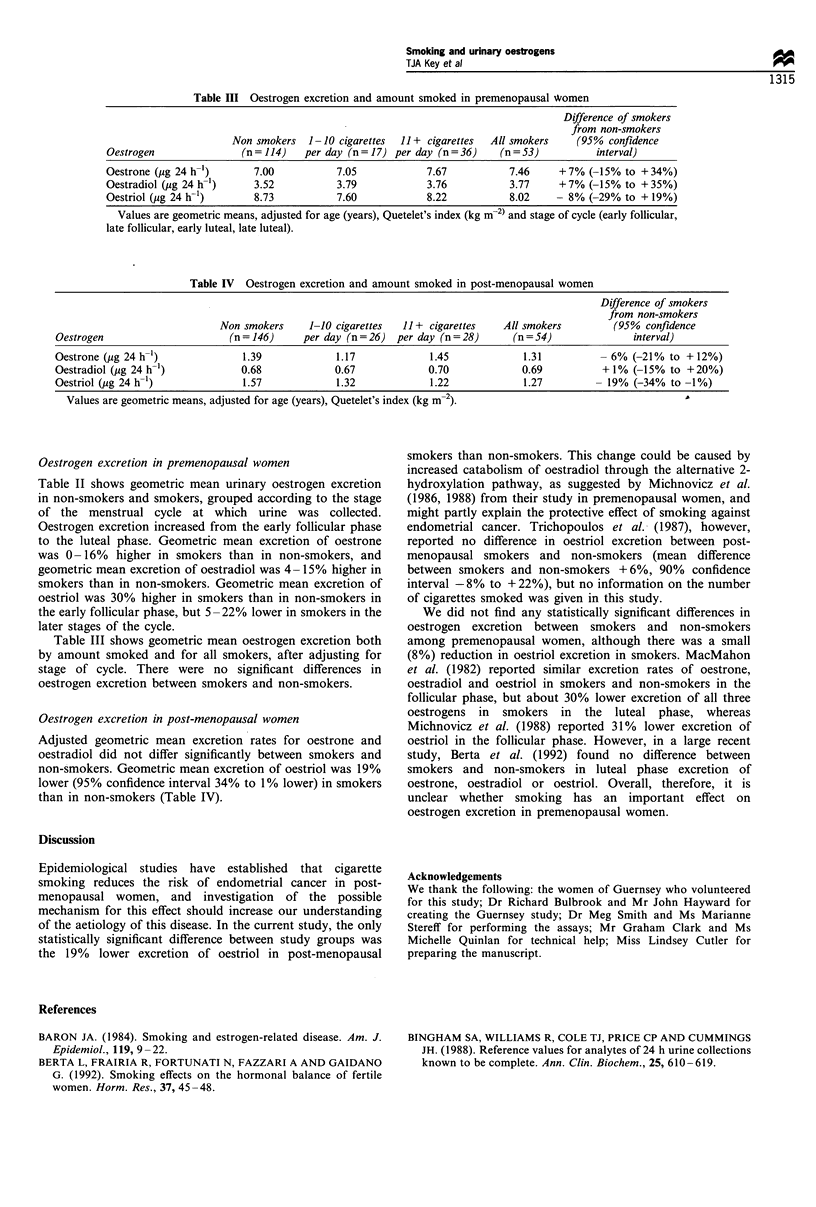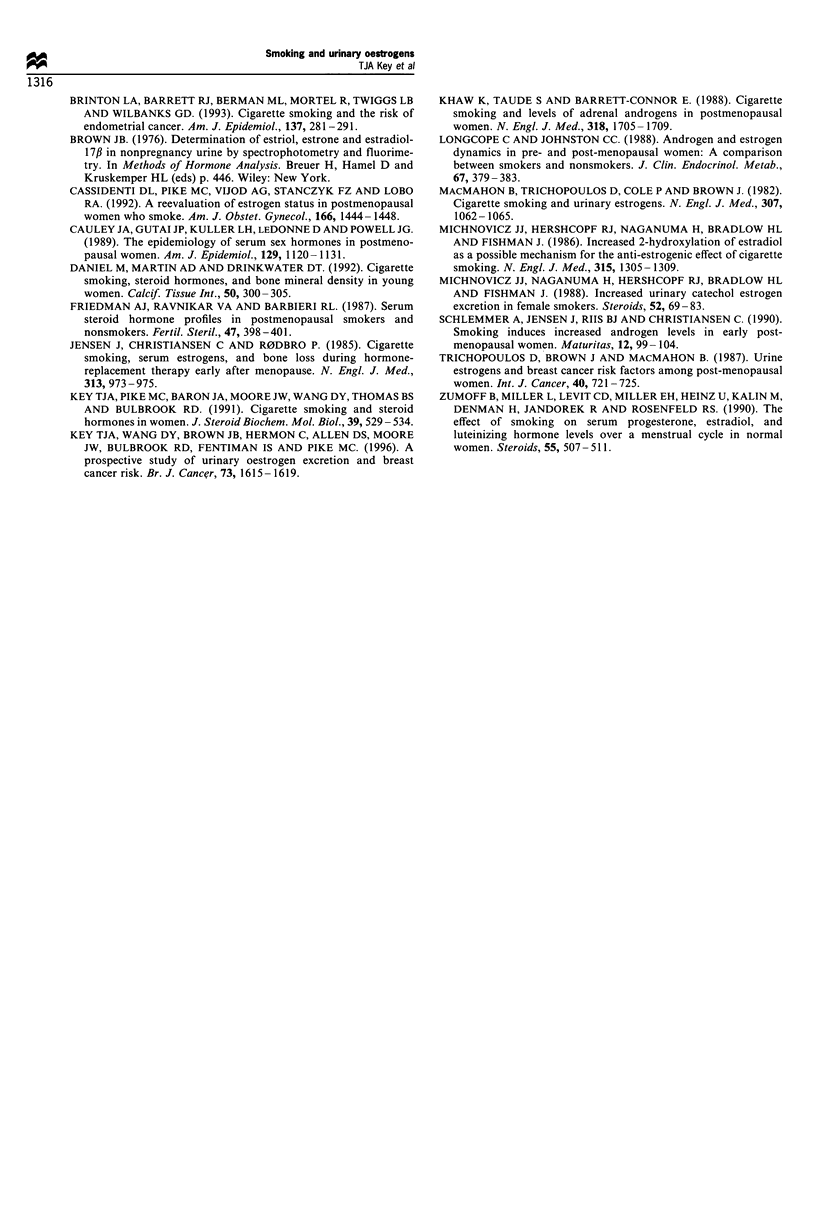# Cigarette smoking and urinary oestrogen excretion in premenopausal and post-menopausal women.

**DOI:** 10.1038/bjc.1996.536

**Published:** 1996-10

**Authors:** T. J. Key, M. C. Pike, J. B. Brown, C. Hermon, D. S. Allen, D. Y. Wang

**Affiliations:** Imperial Cancer Research Fund, Radcliffe Infirmary, Oxford, UK.

## Abstract

Cigarette smoking is associated with a reduction in the risk for endometrial cancer in post-menopausal women and it has been suggested that this is because smoking has an anti-oestrogenic effect. To investigate this, concentrations of oestrone, oestradiol and oestriol were measured in 24 h urine samples from 167 premenopausal women (53 smokers, 114 non-smokers) and 200 post-menopausal women (54 smokers, 146 non-smokers). Among premenopausal women there were no significant differences in oestrogen excretion between smokers and non-smokers. Among post-menopausal women, geometric mean excretion rates for oestrone and oestradiol did not differ significantly between groups, but oestriol excretion was 19% lower (95% confidence interval -34% to -1%) in smokers than in non-smokers. This may partly explain the reduced risk for endometrial cancer among post-menopausal smokers.


British Journal of Cancer (1996) 74, 1313-1316
© 1996 Stockton Press All rights reserved 0007-0920/96 $12.00

### Cigarette smoking and urinary oestrogen excretion in premenopausal and post-menopausal women

TJA Key[1], MC Pike[2], JB Brown[3], C Hermon[1], DS Allen[4] and DY Wang[5]

[1]*Imperial Cancer Research Fund, Cancer Epidemiology Unit, Gibson Building, Radcliffe Infirmary, Oxford OX2 6HE, UK;* [2]*University of Southern California School of Medicine, Department of Preventive Medicine, Parkview Medical Building, A201, 1420 San Pablo Street, Los Angeles, California 90033-9987, USA;* [3]*Department of Obstetrics and Gynaecology, University of Melbourne, Parkville, Victoria 3052, Australia;* [4]*Imperial Cancer Research Fund, Lincoln's Inn Fields, London WC2A 3PX, UK;* [5]*Unit of Metabolic Medicine, Chemical Pathology and Clinical Endocrinology, St Mary's Hospital Medical School, London W2 1PG, UK.*

**Summary** Cigarette smoking is associated with a reduction in the risk for endometrial cancer in post-menopausal women and it has been suggested that this is because smoking has an anti-oestrogenic effect. To investigate this, concentrations of oestrone, oestradiol and oestriol were measured in 24 h urine samples from 167 premenopausal women (53 smokers, 114 non-smokers) and 200 post-menopausal women (54 smokers, 146 non-smokers). Among premenopausal women there were no significant differences in oestrogen excretion between smokers and non-smokers. Among post-menopausal women, geometric mean excretion rates for oestrone and oestradiol did not differ significantly between groups, but oestriol excretion was 19% lower (95% confidence interval −34% to −1%) in smokers than in non-smokers. This may partly explain the reduced risk for endometrial cancer among post-menopausal smokers.

**Keywords:** cigarette smoking; urinary oestrogen; endometrial cancer risk; epidemiology

In comparison with non-smokers, women who smoke have a lower risk of post-menopausal endometrial cancer, an earlier menopause and a higher incidence of osteoporosis, and it has been suggested that all these associations are consequences of an anti-oestrogenic effect of cigarette smoking (Baron, 1984). The protective effect against post-menopausal endometrial cancer is substantial; for example, in a large population-based case–control study, Brinton *et al.* (1993) reported a relative risk of 0.4 in current smokers *vs* never smokers. Although this finding is of no direct value in preventing endometrial cancer, understanding of the mechanism involved is important and a number of studies have therefore investigated the association of cigarette smoking with sex hormones.

MacMahon *et al.* (1982) reported that premenopausal women smokers have reduced urinary excretion of oestrone, oestradiol and oestriol in the luteal phase (but not in the follicular phase) of the menstrual cycle and suggested that this might be caused by reduced oestrogen production. Subsequent investigations have shown that the relationship of cigarette smoking with steroid hormones is complex. Several studies have reported that smoking increases adrenal activity, resulting in increased serum concentrations of androstenedione and dehydroepiandrosterone sulphate, and of progesterone in post-menopausal women (and in the early follicular phase) (Friedman *et al.*, 1987; Schlemmer *et al.*, 1990; Zumoff *et al.*, 1990; Cassidenti *et al.*, 1992). Smoking is not associated with serum concentrations of endogenous oestradiol among premenopausal or post-menopausal women (Friedman *et al.*, 1987; Khaw *et al.*, 1988; Longcope and Johnston, 1988; Cauley *et al.*, 1989; Zumoff *et al.*, 1990; Key *et al.*, 1991; Berta *et al.*, 1992; Cassidenti *et al.*, 1992; Daniel *et al.*, 1992). These results suggest that the protective effect of smoking might be due to the increase in androgens and/or progesterone rather than due to a reduction in oestrogen exposure, although other investigations suggest that smoking does alter the metabolism of oestradiol. Serum concentrations of oestradiol are lower in smokers than in non-smokers among post-menopausal women taking oral oestrogen replacement therapy (Jensen *et al.*, 1985), and Michnovicz *et al.* (1986, 1988) reported that the proportion of estradiol metabolised by 2-hydroxylation is significantly higher in premenopausal women who smoke than in non-smokers and is accompanied by increased excretion of 2-hydroxyoestrone and decreased excretion of oestriol. Michnovicz *et al.* (1986, 1988) suggested that this decrease in the proportion of oestradiol catabolised to oestriol and increase in the proportion catabolised to 2-hydroxyoestrone would cause a net reduction in oestrogenic stimulation and could, therefore, explain the protective effect of smoking against endometrial cancer. However, other studies have failed to find significant differences in urinary oestrogen excretion between smokers and non-smokers among either premenopausal or post-menopausal women (Trichopoulos *et al.*, 1987; Berta *et al.*, 1992). The purpose of the current study was to re-examine the possible association of cigarette smoking with urinary oestrogen excretion and, in particular, to test the hypothesis that smoking is associated with a reduction in the excretion of oestriol (Michnovicz *et al.*, 1988). We were unable to test the hypothesis of increased excretion of 2-hydroxyoestrone in smokers because our assay for this catecholoestrogen was not sufficiently sensitive.

### Materials and methods

### *Subjects*

Between 1977 and 1984, approximately 5000 women aged 34 years and above were recruited into a prospective study of hormones and breast cancer in Guernsey. Height and weight were measured and a questionnaire was completed at interview with details of reproductive history, menopausal status and use of oral contraceptives and hormone replacement therapy. A 24 h urine sample was also collected. In premenopausal women this sample was collected irrespective of the stage of the menstrual cycle, but the dates of onset of menses preceding and following urine collection were recorded. A questionnaire on cigarette smoking was completed by approximately the first 1000 women, recruited between March 1977 and November 1978.

The samples selected for assay of urinary oestrogens were those for all women who were current smokers, were not using exogenous sex hormones, and were either premeno-

Correspondence: TJA Key
Received 5 February 1996; revised 2 May 1996; accepted 8 May 1996

pausal or post-menopausal (natural menopause or bilateral ovariectomy). For premenopausal women, an additional selection criterion was that urine samples had been collected either between 3 and 11 days after the onset of the last menstruation (follicular phase) or between 11 and 3 days before the onset of the next menstruation (luteal phase). Samples for comparison were from women who were known to be non-smokers at recruitment but who met the other criteria, randomly sampled to give a ratio of non-smokers to smokers of approximately 2 to 1 among premenopausal women and approximately 3 to 1 among post-menopausal women.

### *Assays*

Urine samples were considered to be incomplete if the 24 h urine volume was less than or equal to 633 ml, the lower limit of the 95% reference interval in 51 women studied by Bingham *et al.* (1988). Aliquots of urine, identified by code numbers, were sent frozen to the University of Melbourne, where urinary concentrations of oestrone, oestradiol and oestriol were measured during 1989 and 1990 using a method involving spectrophotofluorimetry and internal radioactive standards (Brown, 1976). Assay variation was assessed by including one quality control sample in each run of 12 samples. The mean values and coefficients of variation for this sample were: oestrone 9.8 $\mu g\ l^{-1}$, 11%; oestradiol 3.7 $\mu g\ l^{-1}$, 17%; oestriol 7.2 $\mu g\ l^{-1}$, 14%. These coefficients of variation incorporate both within-assay and between-assay variability. Daily oestrogen excretion was calculated from the concentration in the urine and the volume of urine collected. The sensitivity of the method was sufficient to estimate concentrations as low as 0.1 $\mu g\ l^{-1}$, and no samples were below this limit.

To assess whether there was evidence for deterioration of the samples with long-term storage, we examined the association of total oestrogen excretion (oestrone + oestradiol + oestriol) with the year of urine collection in these samples plus other samples from the same cohort, which were collected later and were assayed for analysis in a nested case–control study of oestrogen excretion and breast cancer risk (Key *et al.*, 1996). There was a statistically significant trend of higher oestrogen excretion in the more recently collected samples, with estimated increases of 6.6% per year and 7.8% per year between 1977 and 1984 in premenopausal and post-menopausal women respectively. This effect will not influence the comparison of smokers with non-smokers because all the samples (smokers and non-smokers) were collected between March 1977 and November 1978 and all the assays were conducted, with samples in arbitrary order, during 1989 and 1990, so that differences in storage time between smokers and non-smokers are minimal.

### *Statistical analysis*

Oestrogen excretion rates were logarithmically transformed to produce approximately normal distributions. Mean oestrogen values presented are geometric means, adjusted by analysis of covariance for age (years) and Quetelet's index (kg $m^{-2}$) and, among premenopausal women where stated, for stage of menstrual cycle using three indicator variables to specify 3–7 and 8–11 days from the beginning of the cycle (early and late follicular) and 11–8 and 7–3 days from the end of the cycle (early and late luteal). To summarise the differences between smokers and non-smokers, we calculated the geometric means (and 95% confidence intervals) of the ratios of the variables as an estimate of the percentage differences between the two groups.

### Results

### *Subject characteristics*

Among premenopausal women, smokers were on average 0.9 years younger and 0.5 kg $m^{-2}$ thinner than non-smokers (Table I). Among post-menopausal women, smokers were on average 2.1 years younger, 0.8 years younger at menopause, and 0.3 kg $m^{-2}$ thinnner than non-smokers (Table I).

**Table I** Characteristics of non-smokers and smokers

| *Variable* | *Non-smokers* Mean | s.d. | n | *Smokers* Mean | s.d. | n | P[a] |
|---|---|---|---|---|---|---|---|
| *Premenopausal* | | | | | | | |
| Age (years) | 42.4 | 4.0 | 114 | 41.5 | 4.8 | 53 | 0.226 |
| Quetelet's index (kg $m^{-2}$) | 24.6 | 3.4 | 114 | 24.1 | 3.8 | 53 | 0.318 |
| *Post-menopausal* | | | | | | | |
| Age (years) | 57.9 | 6.3 | 146 | 55.8 | 6.4 | 54 | 0.042 |
| Age at menopause (years) | 48.7 | 5.3 | 142 | 47.9 | 4.1 | 53 | 0.372 |
| Quetelet's index (kg $m^{-2}$) | 25.3 | 3.5 | 146 | 25.0 | 3.5 | 54 | 0.686 |

[a]Two-sided test for difference between means.

**Table II** Oestrogen excretion and stage of menstrual cycle in premenopausal non-smokers and smokers

| *Cycle stage* | *Non-smokers* Geometric mean | (95% CI) | n | *Smokers* Geometric mean | (95% CI) | n |
|---|---|---|---|---|---|---|
| Oestrone ($\mu g$ 24 $h^{-1}$) | | | | | | |
| Early follicular | 4.61 | (3.17–6.71) | 14 | 5.33 | (3.56–7.99) | 12 |
| Late follicular | 7.33 | (5.72–9.39) | 32 | 7.59 | (5.40–10.65) | 17 |
| Early luteal | 7.60 | (6.13–9.44) | 42 | 7.57 | (5.21–11.00) | 14 |
| Late luteal | 7.78 | (5.91–10.24) | 26 | 8.93 | (5.74–13.91) | 10 |
| Oestradiol ($\mu g$ 24 $h^{-1}$) | | | | | | |
| Early follicular | 2.39 | (1.64–3.48) | 14 | 2.61 | (1.74–3.93) | 12 |
| Late follicular | 3.69 | (2.88–4.74) | 32 | 3.86 | (2.74–5.43) | 17 |
| Early luteal | 3.89 | (3.13–4.84) | 42 | 4.04 | (2.77–5.89) | 14 |
| Late luteal | 3.72 | (2.82–4.91) | 26 | 4.27 | (2.73–6.67) | 10 |
| Oestriol ($\mu g$ 24 $h^{-1}$) | | | | | | |
| Early follicular | 4.06 | (2.66–6.21) | 14 | 5.28 | (3.35–8.35) | 12 |
| Late follicular | 7.11 | (5.38–9.41) | 32 | 6.79 | (4.63–9.97) | 17 |
| Early luteal | 11.78 | (9.22–15.03) | 42 | 9.14 | (5.99–13.96) | 14 |
| Late luteal | 12.11 | (8.88–16.51) | 26 | 9.80 | (5.94–16.16) | 10 |

Geometric mean values are adjusted for age (years) and Quetelet's index (kg $m^{-2}$).

Table III Oestrogen excretion and amount smoked in premenopausal women

| *Oestrogen* | *Non smokers (n=114)* | *1–10 cigarettes per day (n=17)* | *11+ cigarettes per day (n=36)* | *All smokers (n=53)* | *Difference of smokers from non-smokers (95% confidence interval)* |
|---|---|---|---|---|---|
| Oestrone ($\mu g$ 24 $h^{-1}$) | 7.00 | 7.05 | 7.67 | 7.46 | +7% (–15% to +34%) |
| Oestradiol ($\mu g$ 24 $h^{-1}$) | 3.52 | 3.79 | 3.76 | 3.77 | +7% (–15% to +35%) |
| Oestriol ($\mu g$ 24 $h^{-1}$) | 8.73 | 7.60 | 8.22 | 8.02 | – 8% (–29% to +19%) |

Values are geometric means, adjusted for age (years), Quetelet's index (kg $m^{-2}$) and stage of cycle (early follicular, late follicular, early luteal, late luteal).

Table IV Oestrogen excretion and amount smoked in post-menopausal women

| *Oestrogen* | *Non smokers (n=146)* | *1–10 cigarettes per day (n=26)* | *11+ cigarettes per day (n=28)* | *All smokers (n=54)* | *Difference of smokers from non-smokers (95% confidence interval)* |
|---|---|---|---|---|---|
| Oestrone ($\mu g$ 24 $h^{-1}$) | 1.39 | 1.17 | 1.45 | 1.31 | – 6% (–21% to +12%) |
| Oestradiol ($\mu g$ 24 $h^{-1}$) | 0.68 | 0.67 | 0.70 | 0.69 | +1% (–15% to +20%) |
| Oestriol ($\mu g$ 24 $h^{-1}$) | 1.57 | 1.32 | 1.22 | 1.27 | – 19% (–34% to –1%) |

Values are geometric means, adjusted for age (years), Quetelet's index (kg $m^{-2}$).

*Oestrogen excretion in premenopausal women*

Table II shows geometric mean urinary oestrogen excretion in non-smokers and smokers, grouped according to the stage of the menstrual cycle at which urine was collected. Oestrogen excretion increased from the early follicular phase to the luteal phase. Geometric mean excretion of oestrone was 0–16% higher in smokers than in non-smokers, and geometric mean excretion of oestradiol was 4–15% higher in smokers than in non-smokers. Geometric mean excretion of oestriol was 30% higher in smokers than in non-smokers in the early follicular phase, but 5–22% lower in smokers in the later stages of the cycle.

Table III shows geometric mean oestrogen excretion both by amount smoked and for all smokers, after adjusting for stage of cycle. There were no significant differences in oestrogen excretion between smokers and non-smokers.

*Oestrogen excretion in post-menopausal women*

Adjusted geometric mean excretion rates for oestrone and oestradiol did not differ significantly between smokers and non-smokers. Geometric mean excretion of oestriol was 19% lower (95% confidence interval 34% to 1% lower) in smokers than in non-smokers (Table IV).

### Discussion

Epidemiological studies have established that cigarette smoking reduces the risk of endometrial cancer in post-menopausal women, and investigation of the possible mechanism for this effect should increase our understanding of the aetiology of this disease. In the current study, the only statistically significant difference between study groups was the 19% lower excretion of oestriol in post-menopausal smokers than non-smokers. This change could be caused by increased catabolism of oestradiol through the alternative 2-hydroxylation pathway, as suggested by Michnovicz *et al.* (1986, 1988) from their study in premenopausal women, and might partly explain the protective effect of smoking against endometrial cancer. Trichopoulos *et al.* (1987), however, reported no difference in oestriol excretion between post-menopausal smokers and non-smokers (mean difference between smokers and non-smokers +6%, 90% confidence interval −8% to +22%), but no information on the number of cigarettes smoked was given in this study.

We did not find any statistically significant differences in oestrogen excretion between smokers and non-smokers among premenopausal women, although there was a small (8%) reduction in oestriol excretion in smokers. MacMahon *et al.* (1982) reported similar excretion rates of oestrone, oestradiol and oestriol in smokers and non-smokers in the follicular phase, but about 30% lower excretion of all three oestrogens in smokers in the luteal phase, whereas Michnovicz *et al.* (1988) reported 31% lower excretion of oestriol in the follicular phase. However, in a large recent study, Berta *et al.* (1992) found no difference between smokers and non-smokers in luteal phase excretion of oestrone, oestradiol or oestriol. Overall, therefore, it is unclear whether smoking has an important effect on oestrogen excretion in premenopausal women.

**Acknowledgements**
We thank the following: the women of Guernsey who volunteered for this study; Dr Richard Bulbrook and Mr John Hayward for creating the Guernsey study; Dr Meg Smith and Ms Marianne Stereff for performing the assays; Mr Graham Clark and Ms Michelle Quinlan for technical help; Miss Lindsey Cutler for preparing the manuscript.

### References

BARON JA. (1984). Smoking and estrogen-related disease. *Am. J. Epidemiol.*, **119**, 9–22.

BERTA L, FRAIRIA R, FORTUNATI N, FAZZARI A AND GAIDANO G. (1992). Smoking effects on the hormonal balance of fertile women. *Horm. Res.*, **37**, 45–48.

BINGHAM SA, WILLIAMS R, COLE TJ, PRICE CP AND CUMMINGS JH. (1988). Reference values for analytes of 24 h urine collections known to be complete. *Ann. Clin. Biochem.*, **25**, 610–619.

BRINTON LA, BARRETT RJ, BERMAN ML, MORTEL R, TWIGGS LB AND WILBANKS GD. (1993). Cigarette smoking and the risk of endometrial cancer. *Am. J. Epidemiol.*, **137**, 281–291.

BROWN JB. (1976). Determination of estriol, estrone and estradiol-17$\beta$ in nonpregnancy urine by spectrophotometry and fluorimetry. In *Methods of Hormone Analysis*. Breuer H, Hamel D and Kruskemper HL (eds) p. 446. Wiley: New York.

CASSIDENTI DL, PIKE MC, VIJOD AG, STANCZYK FZ AND LOBO RA. (1992). A reevaluation of estrogen status in postmenopausal women who smoke. *Am. J. Obstet. Gynecol.*, **166**, 1444–1448.

CAULEY JA, GUTAI JP, KULLER LH, LEDONNE D AND POWELL JG. (1989). The epidemiology of serum sex hormones in postmenopausal women. *Am. J. Epidemiol.*, **129**, 1120–1131.

DANIEL M, MARTIN AD AND DRINKWATER DT. (1992). Cigarette smoking, steroid hormones, and bone mineral density in young women. *Calcif. Tissue Int.*, **50**, 300–305.

FRIEDMAN AJ, RAVNIKAR VA AND BARBIERI RL. (1987). Serum steroid hormone profiles in postmenopausal smokers and nonsmokers. *Fertil. Steril.*, **47**, 398–401.

JENSEN J, CHRISTIANSEN C AND RØDBRO P. (1985). Cigarette smoking, serum estrogens, and bone loss during hormone-replacement therapy early after menopause. *N. Engl. J. Med.*, **313**, 973–975.

KEY TJA, PIKE MC, BARON JA, MOORE JW, WANG DY, THOMAS BS AND BULBROOK RD. (1991). Cigarette smoking and steroid hormones in women. *J. Steroid Biochem. Mol. Biol.*, **39**, 529–534.

KEY TJA, WANG DY, BROWN JB, HERMON C, ALLEN DS, MOORE JW, BULBROOK RD, FENTIMAN IS AND PIKE MC. (1996). A prospective study of urinary oestrogen excretion and breast cancer risk. *Br. J. Cancer*, **73**, 1615–1619.

KHAW K, TAUDE S AND BARRETT-CONNOR E. (1988). Cigarette smoking and levels of adrenal androgens in postmenopausal women. *N. Engl. J. Med.*, **318**, 1705–1709.

LONGCOPE C AND JOHNSTON CC. (1988). Androgen and estrogen dynamics in pre- and post-menopausal women: A comparison between smokers and nonsmokers. *J. Clin. Endocrinol. Metab.*, **67**, 379–383.

MACMAHON B, TRICHOPOULOS D, COLE P AND BROWN J. (1982). Cigarette smoking and urinary estrogens. *N. Engl. J. Med.*, **307**, 1062–1065.

MICHNOVICZ JJ, HERSHCOPF RJ, NAGANUMA H, BRADLOW HL AND FISHMAN J. (1986). Increased 2-hydroxylation of estradiol as a possible mechanism for the anti-estrogenic effect of cigarette smoking. *N. Engl. J. Med.*, **315**, 1305–1309.

MICHNOVICZ JJ, NAGANUMA H, HERSHCOPF RJ, BRADLOW HL AND FISHMAN J. (1988). Increased urinary catechol estrogen excretion in female smokers. *Steroids*, **52**, 69–83.

SCHLEMMER A, JENSEN J, RIIS BJ AND CHRISTIANSEN C. (1990). Smoking induces increased androgen levels in early post-menopausal women. *Maturitas*, **12**, 99–104.

TRICHOPOULOS D, BROWN J AND MACMAHON B. (1987). Urine estrogens and breast cancer risk factors among post-menopausal women. *Int. J. Cancer*, **40**, 721–725.

ZUMOFF B, MILLER L, LEVIT CD, MILLER EH, HEINZ U, KALIN M, DENMAN H, JANDOREK R AND ROSENFELD RS. (1990). The effect of smoking on serum progesterone, estradiol, and luteinizing hormone levels over a menstrual cycle in normal women. *Steroids*, **55**, 507–511.